# First case report of PLA2R-related monotypic (IgG-κ positive) membranous nephropathy concurrent with leukocyte chemotactic factor 2 amyloidosis

**DOI:** 10.1186/s12882-023-03331-x

**Published:** 2023-09-26

**Authors:** Jing Xu, Xinlu Wang, Qinjie Weng, Xiaobei Feng, Xiaoxia Pan

**Affiliations:** 1 Department of Nephrology, Shanghai Ruijin Hospital, Shanghai Jiao Tong University, School of Medicine, Shanghai, China; 2 Department of Nephrology, Institute of Nephrology, 197, Ruijin Er Road, Shanghai, China 200025

**Keywords:** Monotypic, Membranous nephropathy (MN), Phospholipase A2 receptor (PLA2R), Leukocyte chemotactic factor 2 amyloidosis (ALECT2), Case report

## Abstract

**Background:**

Membranous nephropathy (MN) is a major pattern of nephrotic syndrome (NS) in adults. Some MN have secondary causes and some may be accompanied with other glomerular diseases. MN patients coexisting with amyloidosis are very rare, and mostly was polytypic MN. Herein, we describe the first report which identifying monotype PLA2R-MN (κ light chain) concurrent with leukocyte chemotactic factor 2 amyloidosis (ALECT2). This rare case highlights the importance of renal pathology for diagnosis.

**Case presentation:**

We describe a case of a 60-year-old male patient with persistent proteinuria and low serum albumin for nine months. No monoclonal component was revealed by serum and urine immunofixation electrophoresis but serum PLA2R antibody was positive. The patient was empirically treated with Leflunomide and Losartan, but edema was not improved. The diagnosis of renal pathology is PLA2R-related monotypic (IgG-κ positive) MN concurrent with ALECT2. Methylprednisolone, cyclosporine A and anticoagulant (rivaroxaban) were prescribed resulting in a complete remission of NS.

**Conclusions:**

MN patients concurrent with ALECT2 presented massive proteinuria or NS. When nephrotic range proteinuria is present in ALECT2, it is important to consider that it may be due to a concomitant underlying nephropathy especially MN and treated according to MN will get good therapeutic effect.

## Background

In recent years, the incidence rate and prevalence of membranous nephropathy (MN) have shown an upward trend, accounting for about 6.0% ~ 28.8% of primary glomerular diseases [1]. Approximately 80% of MN cases have no clear secondary causes, referred to as idiopathic membranous nephropathy (IMN) or primary membranous nephropathy (PMN), and approximately 20% of MN cases are secondary to autoimmune diseases, infections, malignancies, drug use, and heavy metal poisoning, referred to as secondary membranous nephropathy (SMN). Phospholipase A2 receptor (PLA2R) was the major target antigen described in MN and account for 70–80% of IMN cases. Thrombospondin type 1 domain-containing7A(THSD7A), semaphorin 3B (SEMA 3B), neural EGF-like 1 protein (NELL1), and protocadherin 7 (PCDH7) et al. are more recently described target antigens [2]. Cases of MN combined with amyloidosis have been demonstrated rarely. We herein report a case of PLA2R-positive monotypic MN with leukocyte chemotactic factor 2 amyloidosis (ALECT2) featuring NS.

## Case presentation

A 60-year-old male was admitted to our hospital for persistent proteinuria for nine months. Proteinuria, microscopic hematuria, and renal function involvement (serum creatinine, Scr 1.66 mg/dL) were detected in May 2013 with abdominal pain and urinary calculi found by ultrasound. Scr decreased to normal (0.98 mg/dL) two days later. Foamy urine was noticed by himself for five months and periorbital edema for three months. 24-h proteinuria was ranging from 9.8 g to 11.48 g since Nov 2013 with Serum albumin 2.37 g/dL. The patient was empirically treated with Leflunomide, Losartan and Chinese traditional herbs. However, edema was not improved. No history of hypertension, diabetes mellitus, coronary heart disease and other chronic diseases. His body weight lost 4 kg within four months before hospitalization.

Physical examination at admission: blood pressure 114/69 mmHg, temperature 36.3 °C, heart rate 60 bpm, body weight 82 kg, minor periorbital edema was noted. Laboratory test revealed Scr 0.89 mg/dL with estimated glomerular filtration rate (eGFR) of 93.3 mL/min/1.73m^2^ calculated by EPI equation, serum albumin 1.5 g/dL, serum triglyceride 589 mg/dL (2.21 mmol/L), serum total cholesterol 191.2 mg/dl (10.62 mmol/L) and hemoglobin 13.9 g/dL. The urinary red blood cell 11–15/HP. 24-h proteinuria 12.5 g/1.2L. Serum IgG 341 mg/dL, IgA 266 mg/dL, IgM 64 mg/dL, C3 118 mg/dL, C4 35 mg/dL. No monoclonal component was revealed by serum and urine immunofixation electrophoresis. Liver function was normal. Kidney size and echo texture was within normal range by ultrasound. Left atrial enlargement was found by cardiac ultrasound, EF normal. Bone marrow aspiration showed low percentage of plasma cells with Congo red staining negative and the ratio of serum free light chain κ/λ was normal. Serum PLA2R antibody was positive using indirect immunofluorescent testing.

**Fig. 1:**
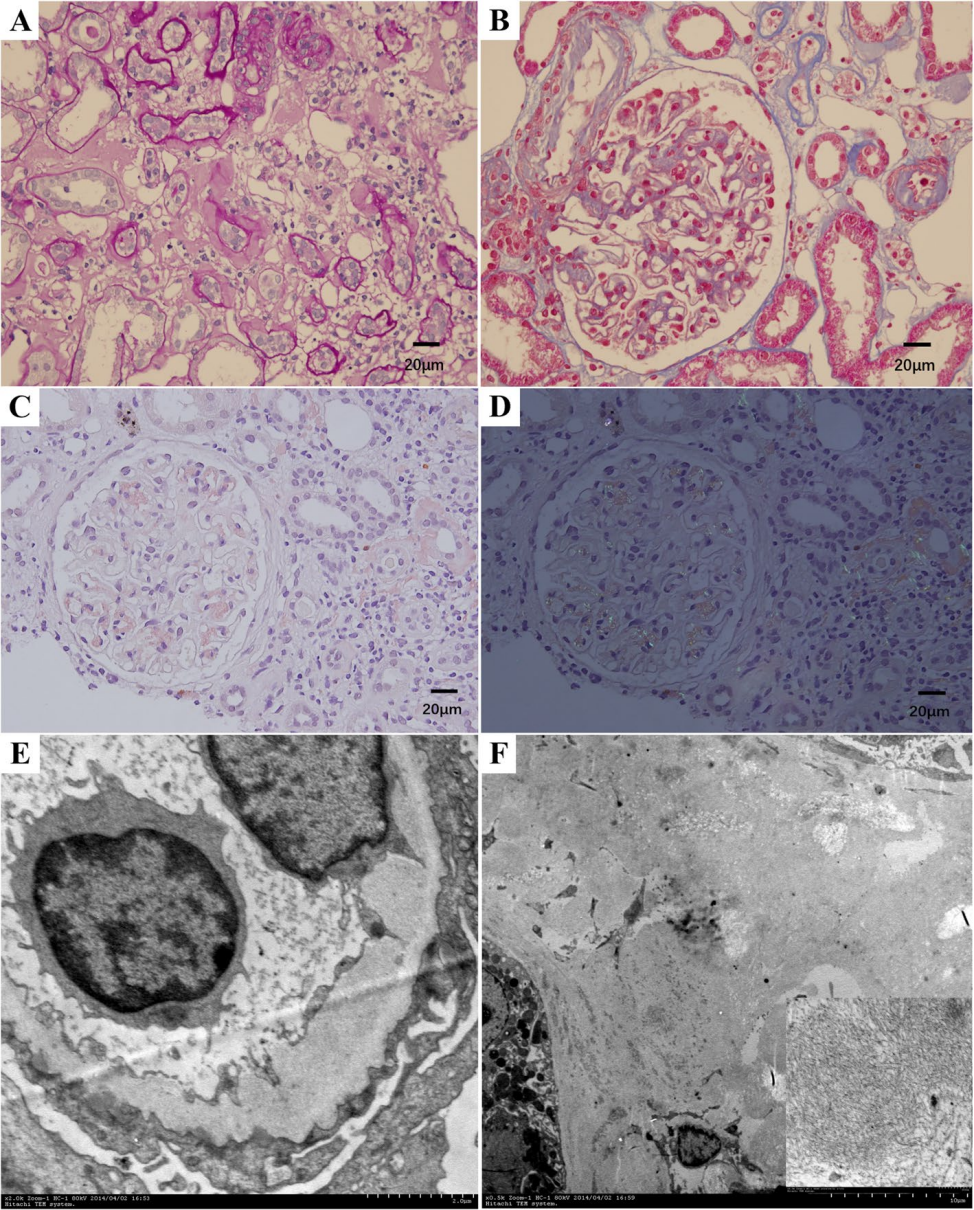
Light and Electron micrographs of biopsy kidney specimen from the patient. **A** Amyloids deposit in the interstitium. Periodic Acid-Schiff stain × 400, **B** Amyloids deposit in afferent arteriole and some mesangial area. Erythrophilic complex deposits on the epithelial side. Masson stain × 400, **C**, **D** Congo red-positive amyloid deposits, with apple-green birefringence, seen in the mesangial area and interstitium. **C **Congo red × 400, **D** Congo red under polarized light microscopy × 400, **E** Subepithelial electron-dense deposits with intervening “spikes” and Amyloid fibrils found in subendothelial spaces. EM × 30,000, **F** Amyloid fibrils found in interstitial area. EM × 7500/ × 90000

Then, he was performed renal biopsy. The entire sample contained 20 glomeruli contained 3 global sclerosed and 1 segmental sclerosed by light microscopy. Mild and diffuse thickening of the glomerular capillary walls with small spikes were seen in segmental capillary loop by methenamine silver stain. Mesangial cellularity was normal. A few glomeruli displayed Congo red-positive amyloid deposits in some small mesangial, with apple-green birefringence, also in interstitium, interlobular arteries and afferent arterioles (Fig. 1A, B, C, D). Lesions of chronic renal tubulointerstitial were mild.

**Fig. 2:**
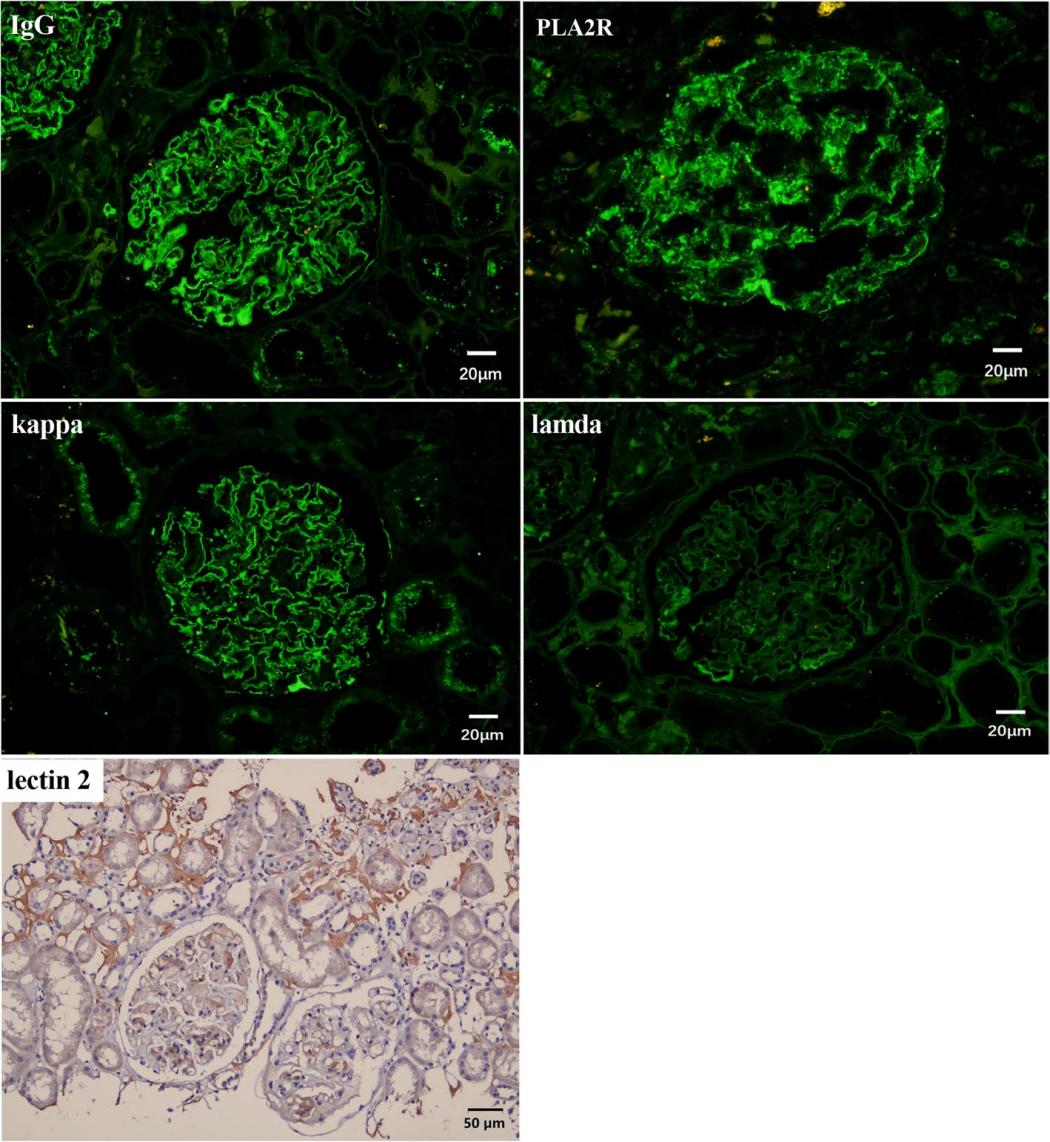
Immunofluorescenct/immunohistochemistry micrographs of the biopsy kidney. Diffuse granular deposition of IgG, κ light chain and anti-PLA2R antibody along the glomerular basement membrane (GBM). λ light chain deposition was seen weakly in interstitium and not seen along the GBM. LECT2 was stained predominantly on amyloid deposits by immunohistochemistry. IgG × 400, PLA2R1 × 400, Light chain κ × 400, Light chains λ × 400, LECT2 × 200

Immunofluorescence revealed diffuse continuous intense (3 +) granular IgG with monotypic light chain κ positive and C3 staining of the glomerular basement membrane (GBM) (Fig. 2). IgG subgroup staining showed codominance of IgG4 (3 +) and IgG1 (trace). Staining for anti-PLA2R antibody showed diffuse granular deposition along the GBM as well (Fig. 2). However, LECT2 was stained predominantly on amyloid deposits by immunohistochemistry (Fig. 2). Light chain λ was trace on the same area, while light chain κ was negative. Staining for amyloid A protein was not observed in the interstitium and mesangium.

Electron microscopy demonstrated diffuse subepithelial electron-dense deposits with intervening “spikes” (Fig. 1E). Amyloid fibrils were found in a few mesangial areas, subendothelial spaces, and interstitial area (Fig. 1F). The diameter of these fibrils was 9.2–13.0 nm.

A diagnosis of PLA2R-related monotypic (IgG-κ positive) membranous nephropathy coexisting with ALECT2 disease was made by renal biopsy. No mutation has been detected in the LECT2 gene in our case. The patient was treated with oral methylprednisolone (initial dosage 16 mg/d) and cyclosporine A (initial dosage 50 mg q12h) for 18 months and nephrotic syndrome (NS) was relieved. After discontinuation of glucocorticoids and cyclosporine for two years, 24-h proteinuria increased to 0.8–1.0 g, and serum albumin decreased to 3.0 g/dL. The patient returned to low-dose glucocorticoid (methylprednisolone 12 mg/d) combined with cyclosporine A (75 mg/d). Methylprednisolone decreased to 2.5 mg/d and cyclosporine A 25 mg qod for maintenance till now. Chinese traditional herbs and anticoagulant (rivaroxaban) were used for a long term during the course of the disease. From 2014–2022,the patient was followed up for 8 years, urine protein excretion decreased to 0.4 g and serum albumin increased to 3.88 g/dL with normal Scr (1.03 mg/dL) on Mar 6th 2022.

## Discussion and conclusions

The case that MN combined with amyloidosis has been seldom reported. Bohle A. et al. showed the first case of perimembranous glomerulonephritis combined with glomerular amyloidosis in 1978 [3]. In 1991, Varshavskiĭ VA et al. reported 15 kidney biopsies from 13 patients with a combination of MN and Amyloid A (AA) amyloidosis. This combination is not considered as a special type of amyloidosis but as a peculiar feature of membranous nephropathy [4]. Since 2012, Chen H et al. [5] and Gao YM et al. [6] reported one Chinese patient with MN and systemic amyloidosis respectively. In the latter case, renal PLA2R was positive. Unfortunately, three Chinese cases all failed to identify the type of amyloid. Furthermore, MN combined with AL amyloidosis was also reported. Antoine M et al. reported one case which showed AL amyloidosis (λ light chain) associated with THSD7A-related MN by renal biopsy, instead of PLA2R-related MN. While, serum immunoelectrophoresis showed detectable but non-quantifiable monoclonal IgG and IgM λ spikes (< 1 g/L) with a normal κ/λ ratio, and urine immunoelectrophoresis revealed the presence of small amounts of IgG λ and free λ light chains [7]. In rare amyloidosis, MN also found in few cases. Among 253 hereditary apolipoprotein A-I (ApoA-1) amyloidosis, MN was found in a patient in an Italy study [8].

Leukocyte chemotactic factor 2 (LECT2) -associated amyloidosis (ALECT2) was first reported in 2008 with a distinct geographic and ethnic preponderance. The most common clinical feature of ALECT2 was progressive renal failure, which often combined with diabetes or hypertension. Approximately 33% patients do not have significant proteinuria [9]. No relevant mutation has been detected in the LECT2 gene, nor in our case. The homozygous for the G allele at nucleotide 172 reported in some ALECT2 patients was not found in our case as well. According to the report, ALECT2 could also be associated with other glomerular diseases, including MN [10]. In recent years, several cases of MN combined with ALECT2 were caught attention as our case. We searched the PubMed database for relevant studies using the following search term: “membranous nephropathy” with “ALECT2”, only 12 cases were identified. In 2014, Samar M. Said et al. [9] and CP Larsen et al. [10] found that MN was respectively present in 2.8% (2/72) and 12.5% (5/40) ALECT2 patients by renal biopsy. In 2020, Dan-yang Li et al. reported MN in Chinese ALECT2 patients accounted for 57.1% (4/7), among them 2 patients’ serum and tissue PLA2R were positive [11]. Li Jia et al. reported an ALECT2 patient coexisting with PLA2R-mediated MN in 2021 [12]. All patients’ information was listed in Table 1. Of which, only 6 patients had detailed information who were all Chinese. Most of them were over 60 years old, combined with hypertension and normal renal function, who presented higher proteinuria and higher frequency of NS than isolated ALECT2 patients. When nephrotic range proteinuria is present in ALECT2 patient, it is important to consider that it may be due to a concomitant underlying nephropathy especially MN [10]. PLA2R in their renal tissue were all positive. It is highly possible that these few patients represent a coincidence of two disease processes. To date, there has been no study to explain whether the coexistence of ALECT2 and MN is pathogenetically related or only incidental, which needs to be further investigated [11]. Otherwise, ALECT2 can be found in association with other types of amyloidosis like immunoglobulin λ light chain amyloidosis, because of which immunofluorescence showed that λ light chain stained predominantly on amyloid deposits, leading to misdiagnosis [10]. In our case, weak λ light chain deposits were also found in the LECT2 amyloid area. Based on immunofixation electrophoresis, serum free light chain and clinical manifestation, we prefer to think that the λ light chain was nonspecifically deposited at the site of LECT2 amyloid.

**Table 1:** Clinical features of MN patients coexisting with LECT2 -associated amyloidosis

Article	Case	Sex	Age	Hypertension	DM	Scr (umol/L)	eGFR (mL/min/1.73m^2^)	Alb(g/L)	UTP (g/24 h)	Serum Anti-PLA2R	renal PLA2R1
Samar M. Said et al. 2014 [9]	1–2	/	/	/	/	/	/	/	/	/	/
CP Larsen et al. 2014 [10]	3–7	/	/	/	/	/	/	/	/	/	/
Dan-yang Li et al 2020 [11]	8	F	74	Yes	Yes	118.0	39.2	26.5	11.26	Pos	( +)
Dan-yang Li et al. 2020 [11]	9	F	61	No	Yes	76.0	73.2	22.3	2.44	NA	( ±)
Dan-yang Li et al. 2020 [11]	10	M	79	Yes	No	541.8	7.96	24.7	7.92	Pos	(+ +)
Dan-yang Li et al. 2020 [11]	11	F	61	Yes	No	70.2	80.5	31.4	3.89	NA	( ±)
Li Jia et al. 2021 [12]	12	F	44	Yes	No	Normal	Normal	30.7	3.55	5.22RU/ML	(+ + +)
The present case	13	M	60	No	No	79.0	93.3	15.0	12.50	Pos	(+ +)

*F* female, *M* male, *DM* diabetes mellitus, *Scr* serum creatinine, *Alb* serum albumin, *UTP* urine total protein, *anti-PLA2R* anti-phospholipase A2 receptor antibody, *NA* not available, *Pos* positive, + positive; ± weakly positive

Immune complexes in most MN cases are composed of polytypic IgG. While Best Rocha A and Larsen CP reported 27 MN cases (including repeated renal biopsy in one post-transplant patient) with light chain restricted deposits, only 14.3% (4/28) were λ light chain. 25.9% (7/27) of MN combined with monomorphic IgG deposits were renal PLA2R positive, and all were κ light chain [13]. Debiec H et al. [14] and Ramachandran R et al. [15] respectively showed one renal PLA2R positive MN patient with κ light chain restricted deposits, but the cause of monoclonal presence in MN is unknown. The biopsy-proven renal amyloidosis and concomitant MN remains an exceptional finding, Among the 12 “MN with ALECT2” patients reported so far, 41.7% (5/12) were negative for both k and λ in renal tissue, another 41.7% (5/12) patients were polytypic PLA2R MN (bothκ and λ are positive), the other 2 patients had no specific clinical and pathological data of light chain. Numerous studies have already revealed the sensitivity and specificity of serum PLA2R antibody levels for the diagnosis of IMN are approximately 74.0% and 95.0%, respectively [16] 0.2021 KDIGO guidelines indicated that patients with nephrotic syndrome (NS) and positive anti-PLA2R antibodies may not need renal biopsy for MN diagnosis [17]. In our perspective, renal biopsy remains necessary because renal pathology can provide more information than serum antibodies in certain cases. We diagnosed a unique case of MN in conjunction with monotypic PLA2R (only positive for IgG-k) and concurrent ALECT2 through renal biopsy, but the mechanism is also unclear.

There is currently no specific treatment for ALECT2. Previously reported cases showed that ALECT2 had less or none proteinuria, however, MN patients concurrent with ALECT2 presented massive proteinuria or NS, considering which was more related to MN and treated according to MN [14]. In our case, the patient was mainly treated for MN, achieving remission of NS by the treatment of glucocorticoid and calcineurin inhibitor (cyclosporine A). It is suggested that NS of this patient was more related to PLA2R related MN.

Our study reported the first case of monotype PLA2R-MN (κ light chain) concurrent with ALECT2. The diagnosis was confirmed by renal biopsy. The pathogenesis requires further investigation. Although the blood PLA2R is positive, renal biopsy remains crucial for certain specific cases of MN, as demonstrated in the case we have reported.

## Data Availability

Data sharing is not applicable to this article, as no datasets were generated or analyzed during the current study.

## Abbreviations

MN: Membranous nephropathy
NS: Nephrotic syndrome
PLA2R: Phospholipase A2 receptor
ALECT2: Leukocyte chemotactic factor 2 amyloidosis
IMN: Idiopathic membranous nephropathy
PMN: Primary membranous nephropathy
SMN: Secondary membranous nephropathy
THSD7A: Thrombospondin type 1 domain-containing7A
SEMA 3B: Semaphorin 3B
NELL1: Neural EGF-like 1 protein
PCDH7: Protocadherin 7
Scr: Serum creatinine
eGFR: Estimated glomerular filtration rate
Ig: Immunoglobulin
C3: Complement 3
C4: Complement 4
EF: Ejection fraction
GBM: Glomerular basement membrane
AA: Amyloid A
ApoA-1: Apolipoprotein A-I
LECT2: Leukocyte chemotactic factor 2
